# Work Change in Multiple Sclerosis as Motivated by the Pursuit of Illness-Work-Life Balance: A Qualitative Study

**DOI:** 10.1155/2017/8010912

**Published:** 2017-11-16

**Authors:** Lavanya Vijayasingham, Uma Jogulu, Pascale Allotey

**Affiliations:** ^1^Jeffrey Cheah School of Medicine, Monash University Malaysia, Jalan Lagoon Selatan, Bandar Sunway, Malaysia; ^2^Multiple Sclerosis Society Malaysia, Unit 4-14, 4th Floor, Building Information Centre, Jalan 243, 46100 Petaling Jaya, Malaysia; ^3^School of Business and Law, Edith Cowan University, Joondalup Drive, Joondalup, WA, Australia; ^4^United Nations University-International Institute for Global Health (UNU-IIGH) Building, UKM Medical Centre, Jalan Yaacob Latif, 56000 Kuala Lumpur, Malaysia

## Abstract

Individuals with multiple sclerosis have a tendency to make early decisions for work change, even in reversible, episodic, or mild disease stages. To better understand how a multiple sclerosis (MS) diagnosis influences perceptions of work and motivations for work changes, we conducted a hermeneutic phenomenology study to explore the work lives of ten individuals with MS in Malaysia. The interpretive analysis and cumulative narratives depict an overarching change in their concept of ideal work and life aspirations and how participants make preemptive work changes to manage illness-work-life futures in subjectively meaningful ways. Discussions on their integrated pursuit of finding dynamic and subjective illness-work-life balance include reconciling the problem of hard work and stress on disease activity and progress, making positive lifestyle changes as health management behaviour, and the motivational influence of their own life and family roles: the consideration of their spouses, parents, and children. At an action level, work change was seen as moral and necessary for the management of illness futures. Our findings contribute insights on how individual perceptions and holistic life management decisions contribute to on-going and disrupted work trajectories, which can inform practice and policy on early interventions to support continued employment.

## 1. Introduction

Continued work in individuals with multiple sclerosis (MS) is a necessary disease management goal because work is positively correlated with quality of life, comprehensive symptom and disease management, reduced productivity loss, and reduced need for welfare support. The converse outcomes contribute to the individual and national burden of multiple sclerosis [1–4]. Regrettably, MS is associated with work difficulties, reduction of work hours or participation, work changes such as transitioning from high-stress or demand work roles, and voluntary and involuntary work termination or unemployment [5, 6]. For example, in a global survey of 11,515 of individuals with MS in 2015, approximately 21% of this survey cohort reported becoming unemployed because of MS within three years of diagnosis and 34% within ten years [7]. Past research suggests that these negative work outcomes are linked with increasing age, duration, and severity of illness [8–10]. These studies attribute limiting physical and mental symptoms or their ineffective management as main contributing factors to these work outcomes. Yet, past research also identifies trends of premature work changes or termination, even at early, relapsing, or mild stages or disease and in the absence of permanent limiting functional changes [5, 11]. The motivation and prompting dynamics for these changes are not well understood, and researchers have called for further qualitative research on the relationship between work and MS, particularly at early stages after diagnosis [7].

In this article, we draw attention to how individuals perceive, imagine, and negotiate the long-term course of work after a MS diagnosis. Based on hermeneutic phenomenology study of 10 working individuals with MS in Malaysia, we present themes and discussions of their changing views and approach of work life after a MS diagnosis and MS as a prompt to find intersubjective and dynamic work-life-illness balance. A MS diagnosis introduces a field of new possible futures. On one side, participant discussed the threats of present and future health and functional status, work trajectories, and life roles. On the other was the opposing pursuit to create best versions of the present and future through active management of illness, work, and life. The space in between these fears and goals formed how participants perceived, experienced, and consequently made decisions regarding work. We present three overarching themes, as discussed by participants that influenced and prompted their pursuit of work changes.

## 2. Methods

Hermeneutic phenomenology is the interpretive study of lived and experiential meanings [12]. This qualitative approach was considered appropriate to acquire rich and nuanced knowledge of illness life that is usually experienced and managed within an intersubjective private and personal space.

### 2.1. Participant Access and Selection

Malaysian neurologist estimates a prevalence of 1–3 MS cases per 100,000 people: approximately 900–1000 in a population of 31 million [13, 14]. Given the niche population, access was sought through by key informants within the Multiple Sclerosis Society of Malaysia committee, who made personal introductions and allowed use of the society's social media platform to invite potential participants. The selection criteria for participants included a MS diagnosis, confirmed employment history with intent to continue long-term employment, and the absence of severe cognitive or mental health symptoms. Many patients who responded to us did not meet the inclusion criteria because they were full-time students, home-makers, and unemployed for a long time because of disabilities.

We included ten individuals from a variation of gender, local ethnicity, age, marital status, and duration that lapsed from time of diagnosis to acquire intense representation, rich descriptions, and theoretical depth of their experiences. The participants are all “white-collar” workers, as based on the International Labour Organisation's ISCO-08 classification of major workgroups (1)–(5), which includes professionals to clerical support and sales workers [15].  Table 1 outlines their details. All participants discussed MS-related work decisions and changes. Levels of disease severity are estimated at EDSS < 3 in all but one of the participants, who is a lawyer and who says she sometimes uses a wheelchair to preserve energy (EDSS estimate 3-4).

This study has received approval from Monash University Human Research Ethics Committee (MUHREC). Informed consent was obtained before data collection. Identifying personal and contextual information has been masked to maintain participant anonymity and privacy.

### 2.2. Data Collection and Analysis

This research method emphasizes richness and closeness of engagement with participants. To achieve this, multiple data collection techniques were used. We conducted initial in-depth interviews (1.5–2 hours) and then followed up through intermittent text-based communications (WhatsApp). We also elicited digital images with captions from participants, so they could depict and represent important aspects of their lives and work with MS. The first author (LV) disclosed her own MS diagnosis to participants which provided some degree of ice-breaking and rapport-building dynamics. Through the course of this research, LV accepted a committee position within the society, and her engagement with the wider group of MS patients, caregivers, and health professionals within the MS Society provided research understanding that supplemented this data collection process.

We used an interview guide with topics such as their process of diagnosis and health-seeking behaviours, past work experiences, perceived illness impact, challenges on work life, fears and goals, adaptation and management strategies, and relational dynamics in their work networks. First interviews were conducted between June and December 2015, with on-going interactions through WhatsApp for digital images or photos, and data queries (information clarification) through to December 2016. Interviews were audio-recorded and transcribed. Raw data was tagged with codes and further refined into patterns and categories and then core themes. Interpretive and analytic understanding was achieved through repeated review to maintain closeness and promote complex understanding of the data. Analysis is presented as a co-construction of knowledge, through a descriptive and interpretive voice with direct quotes from participants in italics.

## 3. Findings

### 3.1. Prognosis: The Field of Worst-Case and Best-Case Futures

Most diagnosis stories involved descriptions of symptoms that affected their work lives. A graphic designer began to see the world in shades of grey, “*my vision from the … from the middle of the eye up till the bottom became grey. I could still see but lost the colours.”* A crane operator developed double vision and could not tell “*which *(buttons)* was real, which was fake.”* A corporate executive found her hands faltering to write with a pen and sign documents. When paged for an emergency, a doctor could not run to respond to a newborn who was not breathing*. “They paged me; a baby had come out flat and not crying. I was on a different floor and I couldn't run! I was holding onto the walls; I took the lift down.” *

These events not only reified the potential illness future, but made a strong impact on how they perceived their work futures and subsequently made decisions about career changes. While these relapse events were responsive to acute use of high dose steroids and reversed to various extents, there was a sense that even these intermittent and reversible experiences would one day impact on their key competencies required for a particular role. Colour perception was essential for the graphic designer's creative expression and work output. Even though his initial symptoms were reversed with high dose steroids, he sought to make work changes. Having worked for the same company for ten years, he was able to request a transition into a sales and marketing position. This career change, however, has only made him become aware of new symptoms.* “I have problems with my speech. I lose words in the middle of a sales pitch and it is so embarrassing. Sometimes, it comes and goes. Like, today, I think I'm a bit fine but on some days, I will lose my vocabulary. I will become a bit stupid.”*

The doctor was on track for a specialty training, which required “*steady hands.*” Just before diagnosis, she noticed some lack of strength in her hand which led to “*shaky hands while cannulating and injecting patients.”* This was a direct threat to not only the safety of her patients, but also her career development and progress. Being an early stage medical professional employed by the national public service, MS imposed a long-term concern to the health system's investment in her future training. When she disclosed her illness to various medical departments that interested her, the program leaders were hesitant to include her in their teams and specialty training programs, asking “*what if we train you and you cannot cope*?” She was later transferred to a medical department that involved a lot of administrative paperwork, rather than clinic time. She decided to quit clinical practice at that stage and accepted a position within a medical technology/pharmaceutical company.

The grim generalised prognosis created imagination of a wide spectrum of possibilities. Each described fear for particular circumstances including physical and permanent impairments such as* paralysis, losing mobility, cognitive skills, and vision* and future life outcomes such as* inability to start a family, future work or requiring to stop work, and needing physical care*. Many discussed the future through goals that need to be achieved before the onset of further progress. “*My goal is to make money. Even if I save up, the fear is still there because, like did I save up enough? Knowing MS, you can wake up one day, and … pop, have a relapse or something more permanent.”*

Positive goals included leading a life with as little illness activity as possible, well-managed mental health and coping, preserving ability to work in the long term, ability to start own business as a means to achieve control and agency over their work pace, being able to make smart financial investments to achieve economic stability, being able to travel and pursue work that was personally rewarding, leading a happy, healthy life with loving family, relationships, and friends. The space between these fears and goals formed how participants narrated their perceptions and consequent decisions and actions surrounding work change.

**Figure 1:**
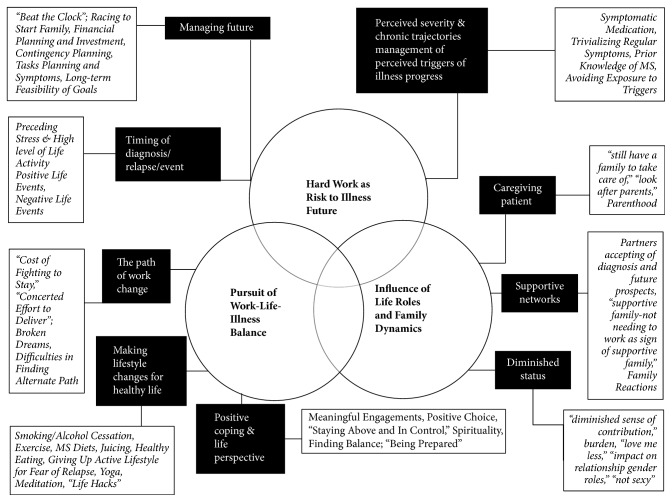
Codes and interpretive theme.

Figure 1 outlines some open and second-level codes, which we interpretively distilled into three integrated themes on the perceptions, experience, and decision-making related to work changes:

1. Hard Work as Risk to Illness Future
2. Pursuit of Illness-Work-Life-Balance
3. The Influence of Life Roles and Family Dynamics on Work Status

### 3.2. Hard Work and Stress as Risk to Illness Future

*Interviewer*

*How would work life be different without MS?*

*Participant*

*Oh, I'll be jumping jobs here and there and playing up the corporate ladder. *

*I love to work. The corporate life … But with MS, I know I cannot handle that much stress. *

*The body cannot handle staying back and working until late hours. You do that and you'll be getting some kind of attack every 6 months. Not worth it. I've got to look after myself. I have to manage this.*

Most other participants discussed the problem of hard work or periods of high life or occupational activity on their personal prognosis or self-perceived rate of illness progress. Participants discussed how stress, both positive (weddings) and negative events (relationship break-ups, job stress, major road accidents, exams, or starting a higher education program), preceded relapses and diagnosis. They discussed perceptions and experience of a link between stress or “hard work” and increased illness activity. Many made statements such as* “The harder you work; the faster the relapse will come”* and “*Don't work too hard, don't stress yourself out. Protect yourself.” *

Most participants also described their ability to* “listen to the body”* and knowing its* “tells.” “Work is very hectic. I fear that it will take a toll on me. This was how I felt- how it was before I got my symptoms *…* I'd get headaches from the stress *…* and then.”* One participant described a previous relapse that happened during a period of high activity in her life. She was able to discern a forthcoming relapse and worked to sort out as much of her responsibilities at work and even handed over projects to colleagues before contacting her neurologist and getting herself admitted for acute treatment. These experiences and perceptions on the consequences of hard, stressful, or high work activity prompted some decisions for work change.

### 3.3. The MS Prompt for Lifestyle Change and the Pursuit of Illness-Work-Life Balance

The pursuit of low illness activity and halted or slow illness progress prompted a stronger pursuit of work-life balance. “*I would like to, have work-life balance; I never gave that much importance before but now that's so important.”* There was the innate sense that the diagnosis prompted them to realign life to prioritize self and loved ones, live, and engage more authentically with their true interests and desires.* “We are so accustomed to being materialistic; we are so accustomed to running after money and status, right? But when you get MS, it makes you realize that life is so much more important. Happiness for me is being healthy.” *

Participants discussed parallel physical, mental, and spiritual lifestyle changes that were prompted by MS and educating themselves more on what may help delay progress and manage their prognosis. Some discussed how MS was a “*kick*” a “*wake-up call” * that induced better life changes and views. “*Actually, I started doing a lot of things that I wanted to do for the past 10 years but I've never got around to it before. So, after MS I told myself, Get off your bum and start doing it.” “If it weren't for MS; I would still be drinking, I would still be smoking but I have not lit a cigarette in years. I would still be sedentary.”*

### 3.4. The Caregiving and Care-Receiving Patient: The Influence of Life Roles and Family Dynamics

* “I may have MS, but I am still a mother.”*

* “I don't want to burden my parents.”*

* “I have to work because I still have a family to take care off.”*

Career evaluations, motivations, and decisions were also tied to the participant's current or aspired future life roles, “*I have to consider who I am taking care of, who is taking care of me.”* This highlights their caregiving and care-receiving roles within their domestic and familial settings. Single participants avidly discussed their need to consider their future and not become a burden on their parents; married participants discussed how they did not want to burden their spouses and families. One of the participants felt that a nonworking woman with MS was the* “sign of a supportive husband and family.*” One participant's mother is also diagnosed with MS and her father with another neurological condition. Since her MS does not limit her, her daily routine involves* “house chores”* and being “*helpful around the house.*” Themes in their discussions revolved around making good life and work-related decisions, to reduce the potentials of future burdens that they may incur and impose on their families and to continue in their caregiving roles within their families.

## 4. Discussion and Conclusion

### 4.1. Work Changes as Health and Life Management Behaviour

Through this study, we observed how a MS diagnosis intersubjectively altered personal concepts of ideal work, existing work goals, and perceptions of work futures. Confluent illness experiences and perceptions, such as hard or stressful work as risk or triggers of increased illness activity and progress, the benefits of lifestyle change to MS futures, and influences of family roles and dynamics, generated holistic illness-work-life goals. Consequently, work changes were discussed as a moral and necessary decision that aligned with overall pursuits of rebalancing these work-illness-life domains.

The quotes and analysis offer a theoretical direction that expands the view from embodied symptoms or functional changes to the longitudinal and subjective course of illness, life, and work. This view prompts a more complex and holistic understanding of how individuals experience and manage a changing health status or make work decisions to manage the risk of future health status deterioration. For instance, the influence of family, domestic and gender roles in the work decisions and outcomes of individuals with MS, and chronic illnesses in general is not well discussed in literature. There is merit in future and further study of these dynamics. The study themes and discussions also underpin process and ability (or inability) of agentic work adaptation and reconstructions.

In this article, we focus on themes that are predominantly embedded in the personal and microlevel of social and economic context. Nevertheless, local context at meso- and macrolevels, such as access to medicines and universal healthcare financing, and various other sociostructural resources and opportunities such as structural protection against health status or disability discrimination in employment also contribute to work decisions and outcomes [16]. In our Malaysian context, we observed that a chronic illness status in employees is often discerned formally and informally through many organizational and human resource management processes, including preemployment medical testing, solicitation of consent to store medical information, administrative paper trails of sick-leave certificates, and applications and usage of employer sponsored medical benefits [17]. These processes often remove the choice of nondisclosure from individuals with chronic illnesses. Such employment practices continue because it is deemed standard practice by the local employment sector, and employment discrimination laws do not deter the practice since the local disability discrimination laws do not include chronic illnesses without permanent impairment [17–19].

Living in the age of the digital and sharing economy, with various review and trust-based platforms and opportunities to monetize online or social media content, we note that past research, including our present study, has not accounted for or captured the effects of these new platforms and changing concepts of work. These new ways and platforms of work prompt new temporalities and pace of work, new contract types, and arrangements with work platforms (rather than employers and workplaces). Individuals may voluntarily terminate traditional forms of formal employment to engage in self-entrepreneurship based on digital, crowd-source, and sharing platforms through fee-for-peer-sharing of resources such as goods, services, and expertise. These new forms of work can confer a continuous source of income, work-life flexibility, control, and agency, in response to work insecurity and challenges. Future quantitative studies and large-scale surveys should consider integrating these trends to explore the influence of changing landscapes and ways of work on the employment trajectories and status of individuals with MS.

In conclusion, key themes from this qualitative study include the integrated pursuit of finding dynamic and subjective illness-work-life balance, which is also influenced by an overarching change in the concept of ideal work and life aspirations. These shifts in perceptions and goals also prompted subjectively meaningful work change as moral and health management action to control illness futures. Further study of these illness-work-life perceptions and motivations in work transitions is necessary for us to better conceptualise, measure, and intervene on continued work participation and protection of individuals with MS. We recommend that future studies adopt a person centric focus on work trajectories with consideration of different life roles and stages, timespans, personal needs, access to resources, individual subjectivities, and social-structural contexts and be inclusive of mild and episodic illness states.

## Figures and Tables

**Table 1:** Demographic details of participants.

	Age	Diagnosed years	Gender	Marital status	Education	Work role	Work type	Industry
(1)	33	3	M	Married	Bachelor's degree	Sales (previous graphic designer)	Full-time	Sales/design

(2)	38	13	M	Married	O-levels equivalent	Promoter (previous machine operator)	Part-time	Sales

(3)	42	18	M	Single	Master's degree	Ph.D. candidate (part-time market research consultant)	Part-time	Market research

(4)	25	8	F	Relationship	Bachelor's degree	Physiotherapist & gym trainer	Full-time	Allied health

(5)	32	6	F	Single	Master's degree	HR/management	Full-time	Petrochemical

(6)	31	5_+_	M	Married	Bachelor's degree	Associate editor (visual)	Shift work	Mass communication

(7)	31	2	F	Relationship	Master's degree	Doctor/medical information specialist	Full-time	Medical/pharmaceuticals

(8)	27	8+	F	Single	A-levels equivalent	Receptionist and author (published fiction novel) and blogger (previous airline staff)	Full-time	Hospitality/publishing/blogging

(9)	46	10	F	Divorced	Master's	Manager	Full-time	Tertiary education

(10)	22	29	F	Married	Master's	Self-employed lawyer	Full-time	Law

O-level equivalent: Sijil Pelajaran Malaysia (SPM), Malaysian Certificate of High School Education. A-level equivalent: Sijil Tinggi Pelajaran Malaysia (STPM), Malaysian Certificate of Higher Education.
